# Individual-Tree Modeling System for Projecting Stem and Heartwood in Clonal Teak Plantations in Eastern Amazon

**DOI:** 10.3390/plants15121890

**Published:** 2026-06-18

**Authors:** Mario Lima dos Santos, Eder Pereira Miguel, Juscelina Arcanjo dos Santos, Gileno Brito de Azevedo, José Natalino Macedo Silva, Cassio Rafael Costa dos Santos, Hallefy Junio de Souza, Leonardo Job Biali, Kennedy Nunes Oliveira

**Affiliations:** 1 Department of Forest Engineering, Federal University of Southern and Southeastern Pará (UNIFESSPA), São Félix do Xingu 68380-000, PA, Brazil; mario.lima@unifesspa.edu.br; 2 Department of Forest Sciences, Faculty of Tecnology, University of Brasilia (UnB), Brasília 70297-400, DF, Brazil; edermiguel@unb.br (E.P.M.); juscelina.santos@unb.br (J.A.d.S.); hallefyj.souza@gmail.com (H.J.d.S.); ljbiali@unb.br (L.J.B.); 3 Department of Forest Engineering, Federal University of Mato Grosso do Sul (UFMS), Chapadão do Sul 79560-000, MS, Brazil; gileno.azevedo@ufms.br; 4 Department of Forest Sciences, Federal Rural University of Amazonia (UFRA), Belém 66077-830, PA, Brazil; silvanatalino734@gmail.com; 5 Department of Forest Sciences, Federal Rural University of Amazonia (UFRA), Capitão Poço 68650-000, PA, Brazil; cassio.santos@ufra.edu.br

**Keywords:** covariate, ITM, rotation, site class, technical age, thinning

## Abstract

Individual tree modeling (ITM) is an effective system for thinned stands, especially in teak (*Tectona grandis* Linn F.) plantations, allowing the estimation of individual-tree-specific variables. Heartwood diameter and volume have high added value and can be estimated in living trees. Therefore, we developed an ITM system for clonal teak stands capable of projecting technical intervention ages and quantifying heartwood production throughout the rotation in the Eastern Brazilian Amazon. The system included equations for total tree height, site index, and taper of both stem and heartwood, with volumes obtained by integrating the respective taper equations. Future diameters and heights were projected using models based on the algebraic difference approach (ADA) and the generalized algebraic difference approach (GADA). Ages of technical intervention were defined by the maximum mean annual increment in volume with bark. The Lundqvist-Korf-ADA base model was the most accurate in estimating future trees’ diameters and heights. The inclusion of the number of trees as a covariate to represent thinning had a significant and positive impact on variable projections. Optimal technical rotations ranged from 17.1 to 21.3 years, considering volume with bark. An increase in the proportion of heartwood was observed, reaching 78% of the diameter and 53% of the volume at rotation ages. The modeling system developed in the present study enables the estimation of technical rotation ages and the quantification of heartwood production throughout the rotation, which provides reliable information for silvicultural planning and decision-making in the management of clonal teak stands.

## 1. Introduction

*Tectona grandis* Linn. f. (teak) is a tree species of high economic and environmental value, widely cultivated in several tropical regions [1]. Although it is one of the most extensively planted species worldwide, the success of its establishment and productivity still faces some challenges, which are mainly associated with the lack of key information to support decision-making regarding stand management. In this context, the modeling of teak growth and yield plays a crucial role in the sustainable planning of commercial forest plantations. Through the application of mathematical models and computer simulations, it is possible to predict tree growth, to estimate timber production over time, and to optimize plantation management [2]. These modeling tools provide valuable insights for forest managers, allowing more accurate decision-making related to several silvicultural aspects, such as tree spacing, stand density, harvest age, and appropriate plantation management practices [3].

Among the projections of forest plantation growth and yield, the modeling system selection depends on the desired level of detail and the management practices to be evaluated [4]. These models can generate projections, such as whole-stand approaches, approaches that consider diameter distribution, as well as individual-tree–level projections [5,6,7]. The latter two categories stand out for providing more detailed estimates of forest growth, including information on the production of multiple timber products, thereby ensuring their maximum utilization [8,9,10]. Such analyses enable more accurate and efficient forest management planning, allowing for proper resource allocation and maximizing forest productivity.

Therefore, individual-tree modeling (ITM) is a crucial approach for thinned stands and for predicting sawn timber production throughout the growth cycle, which is a common aspect of teak plantations. The application of this silvicultural practice makes it possible to account for changes in each tree throughout the rotation, providing essential data to determine the optimal rotation age, as well as the appropriate thinning intensity and timing [11]. For this reason, although teak growth and yield modeling has been predominantly conducted using whole-stand approaches [12,13] and diameter-class models [14,15,16,17], there is a growing need for teak stand growth studies focused on individual-tree modeling.

Additionally, an aspect often neglected in teak growth projections is heartwood production, which significantly adds value. Although significant progress has been made in order to understand the growth and productivity of clonal teak plantations [12], quantitative data on heartwood development and production for living trees—especially throughout the rotation cycle—remain scarce, which hinders decision-making regarding the added value of the final product. Due to the high economic value associated with heartwood, some studies have focused on heartwood formation, variation, and measurement in teak trees, aiming to estimate sawn timber yield and, consequently, ensure its commercial viability [18,19,20,21].

Existing models for estimating teak heartwood are limited to current-state assessment and do not include future projections of heartwood growth and production over time. However, such projections are essential for efficient forest planning and management [22,23,24,25]. The rationale for focusing specifically on heartwood modeling lies in the fact that the commercial value and quality of teak timberwood are determined almost exclusively by the properties of its heartwood, which exhibits high natural durability and resistance to biodeterioration, whereas sapwood has low market value. Therefore, modeling the trees’ internal structure, including heartwood diameter and volume, is essential for quantifying and valuing the final marketable product from trees still standing in the forest [26,27].

Thus, heartwood-related research must be performed together with factors related to its formation, such as silvicultural practices, planting density, tree age, tree height, site class, and genetic material [20,28,29,30]. By considering these production factors and incorporating projections of both tree growth and heartwood development, it is possible to make more accurate and efficient management decisions in teak plantations, ensuring strategic resource maximization and optimizing the commercial value of the timber.

Given this scenario, our study aimed to address the following questions. First question (Q1): What are the optimal rotation ages for thinning and final harvest that maximize outside-bark volume production in clonal teak plantations when projections are performed at the individual-tree level? First hypothesis (H1): Outside-bark volume production is maximized with three thinning interventions carried out at ages of maximum increment, resulting in a final harvest cycle shorter than 25 years. As a second question (Q2), we asked: Is the proposed individual-tree modeling system applicable and efficient for predicting heartwood formation and development in standing teak trees throughout the forest cycle? Second hypothesis (H2): The proposed system is able to predict heartwood formation and development in teak trees during the rotation, thereby allowing the quantification of the added-value structure of teak wood.

Based on these questions and hypotheses, this study aims to develop an individual-tree modeling (ITM) system able to project optimal intervention ages and to quantify heartwood production throughout the rotation of clonal teak plantations in the Eastern Brazilian Amazon.

## 2. Materials and Methods

### 2.1. Study Area and Silvicultural Practices

**Figure 1:**
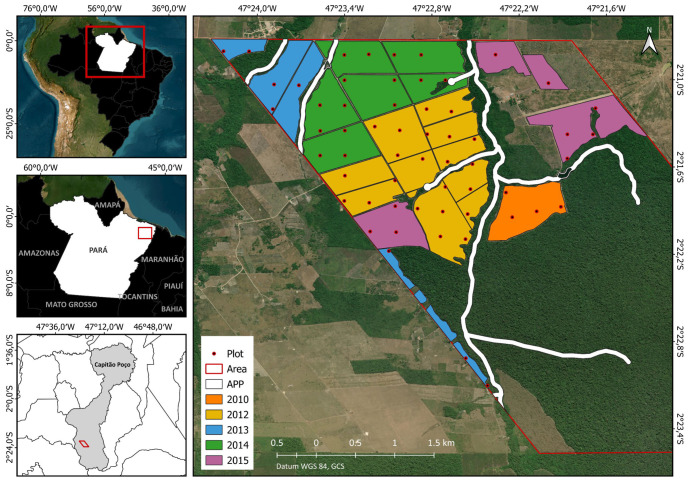
Location of the study area in the municipality of Capitão Poço, state of Pará, Eastern Brazilian Amazon.

The study was conducted in clonal teak plantations established in the municipality of Capitão Poço, located in the state of Pará, in the Eastern Amazon region, Brazil (from 2°30′00″ S; 47°20′00″ W to 2°20′00″ S; 47°30′00″ W) (Figure 1). The topography of the region ranges from gently undulating to flat. The predominant soils are classified as typical dystrophic Yellow Latosols, Plinthic Dystrophic Yellow Latosols, and Concretionary Petric Plinthosols [31]. The native vegetation in this region is primarily dense ombrophilous forest [32]. The climate is classified as Am according to the Köppen classification system and is characterized as hot and humid, with a short dry season [33]. The region has an average annual rainfall of 2256 mm and an average temperature of 26.1 °C [34].

Clonal teak plantations were established in 2010, 2012, 2013, 2014, and 2015. Seedlings were manually planted at spacings of 3.5 m × 3.5 m, 3.75 m × 3.75 m, and 4 m × 4 m. For all stands, the following silvicultural practices were carried out following identical schedules: soil clearing using a bulldozer; control of leaf-cutting ants using ant-killer bait; liming with dolomitic limestone (3 t ha^−1^); planting fertilization with 200 g plant^−1^ of NPK 8-28-16 and 100 g plant^−1^ of KCl applied in the planting pot; weed control through manual hoeing around trees, as well as mechanized and semi-mechanized pruning using a hydraulic tractor; topdressing fertilization with 7 g plant^−1^ of boron and 100 g plant^−1^ of KCl; and manual pruning using a pruning saw and brush cutter [12]. Systematic thinning operations were also carried out at approximately 4.5 (1st) and 8.5 (2nd) years, aiming to reduce the number of trees and basal area by 50% in both thinning interventions [12,13].

### 2.2. Forest Inventory

Annual forest inventories were conducted between 2013 and 2022 on 58 permanent circular plots, for a total of ten consecutive measurement occasions. Each plot presented a 500 m^2^ area. The plots were implanted using systematic samplings, with their respective distribution following a regular grid of 320 m × 320 m (Figure 1). The study area was selected due to the availability of a long-term database derived from these permanent plots in commercial teak plantations, enabling the faithful monitoring of stand growth and production over time. During the inventories, the following variables were recorded and measured: tree age (t), obtained from the occasion of plantation establishment records, diameter at 1.3 m above ground (dbh), measured using diameter tape, total height (ht), measured with a Vertex IV hypsometer [35], and the number of trees removed through thinning.

### 2.3. Individual-Tree Modeling System

Models for projecting future diameter (dbh_1_) and total height (ht_1_) were developed using both the algebraic difference approach (ADA) and the generalized algebraic difference approach (GADA). The base models fitted were: Lundqvist–Korf (M1) and Hossfeld (M2) for ADA, and Cieszewski (M3) and Lundqvist–Korf (M4) for GADA [36,37,38,39] (Table 1). These models were selected since they represent biologically realistic growth functions widely used in forestry modeling. Additionally, they allow a comparison between the ADA and GADA approaches for productive capacity assessment, which differ from each other in their flexibility to represent growth trajectories and site productivity effects. These basic functions have already been used in previous studies conducted in the same teak clonal plantations and in the same region, providing consistent results for the modeling of dendrometric and productivity variables [12,13].

These models were fitted using current diameter (dbh_0_) or total height (ht_0_) values (Y_0_) as a function of current age (t_0_), and future dbh_1_ or ht_1_ values (Y) as a function of future age (t_1_). Models developed under these approaches have been recognized for their ability to accurately represent the main variables influencing forest development, as highlighted by Tewari et al. [36]. These models are based on dynamic functions that exhibit an essential property known as ‘temporal invariance’, as emphasized by Palahí et al. [41]. This temporal invariance is based on the following principle: when projections are made using different initial conditions but the same final conditions, the results obtained are equivalent.

Regression techniques were applied through parameter decomposition and the inclusion of the covariate number of future trees (N_1_, trees ha^−1^). This procedure was performed to represent and incorporate the influence of thinning on the diameter and total height growth of each individual tree in clonal teak stands. For those models that showed the highest accuracy for estimating future diameter with bark (dbh_1_) and future total height (ht_1_), parameter decomposition and the inclusion of the variable N_1_ were performed in order to incorporate the effect of thinning on the growth of these variables. The effect of thinning in individual-tree modeling was represented as a reduction in competition, excluding mortality from the scope of the analysis. Thus, the system did not consider mortality after thinning, fulfilling one of the main goals of this forestry treatment, which is to reduce competition and natural mortality of trees, promoting greater development of the remaining trees.

The choices of tree age as well as the number of trees as explanatory variables were based on biological and operational criteria. Age is the primary determinant of tree growth and the key independent variable in the ADA and GADA models. The number of trees per hectare was selected because it represents stand density and competition among trees, while also reflecting the effects of thinning interventions throughout the stand’s development.

For the calculation of forest variables, a set of individual-tree models was used. First, Equation (1) was used to estimate total tree height for trees that were not directly measured during the forest inventories. This modeling incorporated age as a covariate, derived from the Chapman–Richards sigmoid model [42], allowing accurate estimation of total height. In addition, to determine site index values of 16, 18, and 20 m, Equation (2) was used, based on the algebraic difference approach (ADA) of the Lundqvist–Korf base model, as proposed by Santos et al. [43].

Regarding diameter along the trunk, the Demaerschalk mixed-effects taper model (Equation (3)) was used to estimate diameter at bark (dbw) and heartwood diameter (dh), as proposed by Santos et al. [44]. The heartwood diameter (dh) used in the conicity model was obtained from trunk analysis data, following the methodology described by Santos et al. [44].

The trunk assessment was performed through destructive sampling of 121 trees (trees selected by age, site classes), from which wood disks were removed along the trunk at predefined stem positions using the Hohenadl method. These disks were measured to obtain the diameters with bark, bark-free diameters, and heartwood diameters at different positions along the stem, corresponding to 0, 5, 15, 25, 35, 45, 55, 65, 75, 85, and 95% of the tree’s total height and diameter. Based on these measurements, the longitudinal profile of the heartwood diameter (dh) was reconstructed for each sampled tree, providing the observed data necessary for the calibration and validation of Equation (3).

This model was selected because it showed the best performance among taper functions evaluated for clonal teak plantations in the study region, particularly for estimating diameter distribution and heartwood production. The model was based on the Demaerschalk taper function, with age as a random effect and dummy variables (Tx_1_ and Tx_2_), where Tx_1_ = 0 and Tx_2_ = 0 for diameter with bark, and Tx_1_ = 1 and Tx_2_ = 1 for heartwood diameter, ensuring consistency between diameter estimates.

(1) $$h_{t}=22.35397654\;\cdot \;[1-e^{{\left(-0.05317149\;\;{dbh}_{wb}\right)}^{\left(1.09745889-0.01430881\;t\right)}}]$$

(2) $$SI=24.958604\;\cdot {\left(\frac{dh}{24.958604\;}\right)}^{{\left(\frac{t}{12}\right)}^{0.605413}}$$

(3) $$d_{i}={dbh}_{wb}\left[\sqrt{\;12.40\left(\frac{{\left(t_{h}-h_{i}\right)}^{1.15}}{{t_{h}}^{1.15+1}}\right)+{\;0.73\left(\frac{\left(t_{h}-h_{i}\right)}{t_{h}}\right)}^{30.01}+\;0.02\left(\frac{{\left(t_{h}-h_{i}\right)}^{3.91}}{{t_{h}}^{3.91+1}}\right)}\right]exp\left[\left(-3.34\;Tx_{1}\frac{1}{{dbh}_{wb}}\right)-10.69\;Tx_{2}\frac{1}{{dbh}_{wb}}\right]$$

where d_i_: diameter with bark or heartwood (cm), where diameter corresponds to any height along the stem; dbh_wb_: diameter at breast height with bark (1.3 m above ground); ht: total height (m); h_i_: height at any specified point along the stem (m); dh: dominant height (m); t: age (years); SI: site index (m), where the reference age is 144 months or 12 years. The original models presented the following performance statistics: Equation (1) (RMSE: 1.03 m; RMSPE: 7.98%; AIC: 20,381; $r_{\hat{y}y}$: 0.95); Equation (2); (RMSE: 0.67 m; RMSPE: 4.52%; AIC: 875; $r_{\hat{y}y}$: 0.97); Equation (3) (RMSE: 1.43 cm; RMSPE: 8.05%; AIC: 7382; $r_{\hat{y}y}$: 0.98). The performance statistics refer to the calibration of the original model described by [43,44].

Subsequently, outside-bark total volume (V_wb_) and total heartwood volume (V_h_) were calculated for each tree by integrating their respective taper functions [44]. Finally, using the individual tree volumes obtained, the per-hectare volume was then calculated (m^3^ ha^−1^). This step allows for a more comprehensive and comparative assessment of the productive potential of the forest area under study.

### 2.4. Model Selection and Validation

After models’ fitting for diameter (dbh1) and total height (ht1) prediction, their respective performance was evaluated based on the root mean square error (RMSE), root mean square percentage error (RMSPE) [45], and Akaike Information Criterion (AIC) [46]. The Pearson correlation coefficient between observed and estimated values ($r_{\hat{y}y}$) was used as a complementary measure of association and was not considered a direct indicator of the models’ predictive accuracy. Residual graphical analysis was performed based on simple residuals (observed–estimated), evaluating their distribution regarding the observed and estimated values, as well as through the construction of frequency histograms of relative errors. The significance of regression parameters was also assessed, and residual normality was verified using the Shapiro–Wilk test at a 95% significance level [47].

The dbh1 and ht1 models were fitted using nonlinear generalized least squares through the gnls function from the nlme package in RStudio software, version 4.3.1 [48]. Since the same plots were measured repeatedly over time, the data exhibit a longitudinal structure with potential temporal dependence between successive observations. To account for this intra-plot autocorrelation, the error term was modeled using a first-order continuous autoregressive (AR-1) structure, assuming that the correlation between observations decreases as the time interval between measurements increases [49]. This approach is widely used for longitudinal and unbalanced data and allows for consistent estimates of the parameters and their standard errors in the presence of serial correlation in the residuals [50]. The models were fitted separately under this specification.

The dataset was randomly split into two groups at the plot level: one group used for model fitting (46 plots; 80%, comprising 7662 tree-level observations) and the other group used for model validation (12 plots; 20%, comprising 1992 tree-level observations) (Table 2). The random division preserved the representativeness of the different site classes and planting ages in both subsets, ensuring that the fitting and validation sets encompassed the range of productivity and development conditions observed in the study area.

For model validation, the TOST (two one-sided test) equivalence test based on regression was applied, using a bootstrap approach, since this method is considered one of the most suitable procedures for assessing equivalence between observed and estimated values [9,51]. In the TOST test, the null hypothesis assumes a difference between the observed and estimated values. Thus, rejection of the null hypothesis indicates statistical equivalence between both observed and estimated values. Equivalence was assessed based on the equivalence regions defined for the regression parameters (intercept and slope), using a 99% confidence level and 1000 bootstrap resamples. This test is a statistical procedure used to evaluate whether a model is equivalent to a specific standard or reference [13]. Additionally, a linear regression between observed and predicted values was performed to estimate two confidence limits for the parameters, which were then compared with the estimated equivalence region, as also performed by Santos et al. [12] and Souza et al. [13].

### 2.5. Thinning and Rotation Simulation

Thinning simulations were conducted to evaluate management scenarios in clonal teak plantations in the study region. For this purpose, the studies developed by Santos et al. [12] and Souza et al. [13] were used as references. The regime adopted consisted of basal area removals of 50%, 50%, and 25% in the first, second, and third thinnings, respectively, based on the number of trees. This regime was selected because previous studies conducted under similar site and management conditions indicated that this combination resulted in greater growth and projected production of the stands, in addition to representing a technically viable silvicultural strategy for the region.

The choice of thinning based on the number of trees was deliberate, as this system had already been successfully applied in the study area, using a systematic strip method for tree removal. This thinning approach optimized forest productivity by promoting adequate spacing between the remaining trees, reducing competition, and promoting individual tree growth.

After each simulated thinning, the stand density was updated according to the intensity of the thinning applied. Thus, the volume projections refer exclusively to the remaining stand after each intervention and do not include the volumes removed in previous thinnings.

After obtaining outside-bark and heartwood volume values through the integration of taper equations, volumetric growth increments were calculated to test the first hypothesis and determine the technical thinning (TTA) and rotation (TRA) ages based on maximum outside-bark volume growth. Since these ages were obtained from continuous growth functions, the ITD and ITR values may take on non-integer (decimal) ages, corresponding to the exact mathematical points at which the optimization criteria were met.

The Mean Annual Increment (MAI) was calculated as the ratio of the projected stand volume at age t to the respective age (Equation (4)). The Current Annual Increment (CAI) was calculated as the difference between the projected volumes at two consecutive ages (Equation (5)). Since growth projections were made at annual intervals, the projection interval considered for the CAI calculation was one year. The technical age was defined as the point of intersection between the MAI and CAI curves in volume [52,53]. The second hypothesis was assessed through the full implementation of the individual-tree modeling system, culminating in the calculation of heartwood volume and its respective proportion.

(4) $$MAI=\frac{V_{t}}{t}$$

(5) $$CAI=V_{t}-V_{t-1}$$

where MAI = Mean Annual Increment (m^3^ ha^−1^ yr^−1^); CAI = Current Annual Increment = (m^3^ ha^−1^ yr^−1^); V_t_ = Stand projected volume at age t (m^3^ ha^−1^); V_t−1_ = Stand projected volume at the immediately previous age (m^3^ ha^−1^); t = Stand age (years).

## 3. Results

To select the most accurate regression model in estimating future tree diameter, five different models were assessed, including the model that incorporates the future number of trees per hectare, as a covariate (N_1_) (M5) (Table 3). The accuracy ranking of the regression models without including the covariate was as follows: M1 > M4 > M2 > M3. Based on the comparative analysis, the M1-ADA model proved to be the most accurate among the two approaches (ADA and GADA), showing the smallest prediction errors and the lowest Akaike Information Criterion value. The high correlation coefficient indicated a strong association between the observed and estimated values, corroborating the further performance indicators (Table 3).

Consequently, model M1 was improved by including the future number of trees (N_1_) as a predicting variable, generating model M5, which showed a significant improvement in its ability to explain the variation in tree diameter in thinned stands as age increased. The inclusion of N_1_ was applied to both b_1_ parameters, resulting in an approximately 11% reduction in prediction error compared to the original model form, resulting in RMSE = 0.7723 cm, RMSPE = 4.3150%, and AIC = 15,473.19. The model also presented a high association between observed and predicted values $r_{\hat{y}y}$ = 0.9888. These outcomes reinforce the robustness and accuracy of model M5, turning this model into a suitable choice for estimating future tree diameter at the individual-tree level, especially when considering the thinning effect represented by stand density in the analyzed variable.

The regression model M1-ADA also proved to be a suitable choice for estimating future total tree height (*ht*_1_). In this context, model M5 was developed by incorporating the variable N_1_ in a similar way to that applied for future diameter, resulting in a 1.3% reduction in prediction error compared to the original model. These results indicate that stand density may also influence total height growth, although to a lesser extent than diameter, as stands undergo changes in tree density throughout the rotation. The model presented a high association between observed and predicted values ($r_{\hat{y}y}$ = 0.9509), while maintaining low prediction errors (RMSE = 0.8203 m; RMSPE = 5.6781%) and the lowest AIC value (5335.73) among the evaluated alternatives (Table 4).

**Figure 2:**
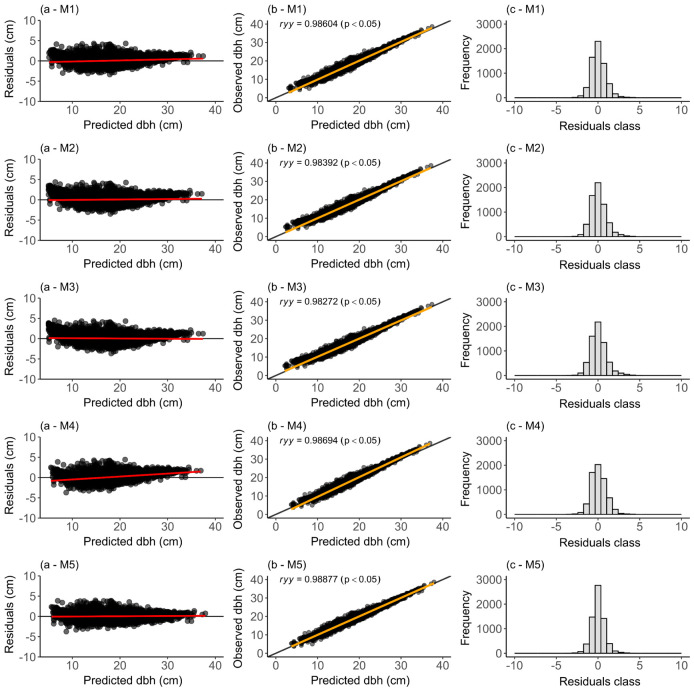
Residual distribution for future diameter at breast height with bark (dbh) (**a**), relationship between observed and predicted diameter with bark values (**b**), and histogram of residual frequency (**c**) for the ADA and GADA models M1–M5 (Table 3) fitted at the individual-tree level in clonal teak plantations in the Eastern Brazilian Amazon. The black points correspond to the observed data, and the red and yellow lines represent trend lines fitted to the scatter plots.

**Figure 3:**
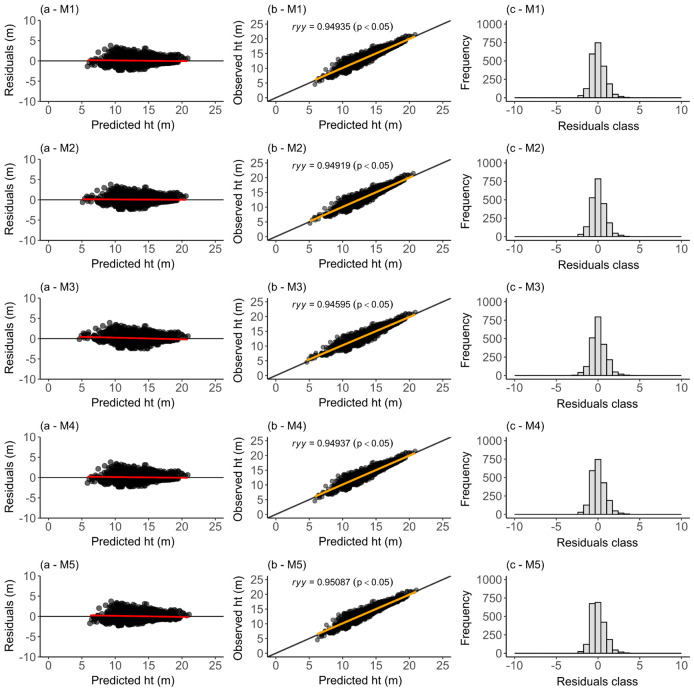
Residual distribution for future total height (**a**), relationship between observed and predicted total height values (**b**), and histogram of residual frequency (**c**) for the ADA and GADA models M1–M5 (Table 4) fitted at the individual-tree level in clonal teak plantations in the Eastern Brazilian Amazon. The black points correspond to the observed data, and the red and yellow lines represent trend lines fitted to the scatter plots.

The residual distribution of the diameter projection models revealed distinct patterns. Model M4, using the GADA, showed overestimation for diameters smaller than 20 cm and underestimation for diameters larger than 20 cm, with residual ranges of ±4.5 cm (Figure 2a). However, the models that were modified by including the covariate (M5), for both diameter and total height, showed no bias in the estimates, with residual ranges of ±3.95 cm and ±3.72 m, respectively (Figure 2a and Figure 3a).

Residual analyses indicated that the modified models (M5) produced unbiased estimates for diameter and total height, exhibiting a homogeneous distribution of errors across the range of observed data. The residuals showed a tendency toward normality, with a higher frequency of observations concentrated in the central error classes (Figure 2c and Figure 3c). Furthermore, the models exhibited lower values of RMSE, RMSPE, and AIC compared to the other evaluated models, demonstrating their superior predictive performance. The high correlation coefficients observed (>0.95) were consistent with these results, indicating a strong association between observed and estimated values.

The projection models for diameter and total height were subjected to an equivalence test for validation. Both models (M5) produced confidence intervals for the model parameters, such as intercept and slope, being into the similarity region. This indicates that the null hypothesis of no difference was rejected for both regression parameters (intercept and slope), confirming the statistical equivalence between the observed and estimated values for diameter and total height and providing independent validation of the selected models, thereby reinforcing their applicability for future projections (Table 5).

Thus, after selecting and validating the most appropriate models for projecting diameter (Equation (6)) and total height (Equation (7)), the Lundqvist–Korf-ADA base equations were adopted, incorporating the covariate *N*_1_, with the aim of composing in order to compose the individual-tree modeling system for clonal teak trees. The resulting equations are presented below:

(6) $${dbh}_{1}\;=\;(b_{1}\;{N_{1}}^{b_{11}}){\left[\frac{{dbh}_{0}}{(b_{1}\;{N_{1}}^{b_{11}})\;}\right]}^{{\left(\frac{t_{0}}{t_{1}}\right)}^{b_{3}}}=\;(1456.51584{\;N_{1}}^{-\;0.55705}){\left[\frac{{dbh}_{0}}{(1456.51584\;{N_{1}}^{-\;0.55705})\;}\right]}^{{\left(\frac{t_{0}}{t_{1}}\right)}^{0.59482\;}}$$

(7) $${ht}_{1}=(b_{1}\;{N_{1}}^{b_{11}}){\left[\frac{{ht}_{0}}{(b_{1}\;{N_{1}}^{b_{11}})\;}\right]}^{{\left(\frac{t_{0}}{t_{1}}\right)}^{b_{3}}}=(66.38997{\;N_{1}}^{-0.18256}){\left[\frac{{ht}_{0}}{(66.38997\;{N_{1}}^{-0.18256})\;}\right]}^{{\left(\frac{t_{0}}{t_{1}}\right)}^{0.78959\;}}$$

where dbh_0_ and dbh_1_ = initial and future diameter at 1.3 m above ground with bark, respectively (cm); ht_0_ and ht_1_ = initial and future total height, respectively (cm); *N*_1_ = future number of trees (trees ha^−1^).

Based on individual-tree level models, projections of diameters and heights were performed for each tree, considering their initial values of diameter at breast height (dbh_0_), total height (ht_0_), and number of trees per unit area (N_0_), at 3 years of age. This projection age was selected due to the presence of an established growth pattern in the stand and the availability of initial measurements for all trees at this age. The plots were stratified according to site class, represented by site indices of 16 m, 18 m, and 20 m.

After the projections, total outside-bark volumes were calculated by integrating the taper equation (Equation (3)). The theoretical growth stagnation and the need for thinning intervention were determined when the mean annual increment (MAI) reached its maximum value, coinciding with the current annual increment (CAI). The mean initial values of dbh_0_, ht_0_, and N_0_ by site class at 3 years of age were also determined. For the 16 m site class, these values were 11.79 cm, 9.63 m, and 784 trees ha^−1^; for the 18 m site class, the values were 13.81 cm, 10.91 m, and 739 trees ha^−1^; and for the 20 m site class, the values were 15.34 cm, 12.05 m, and 709 trees ha^−1^.

**Figure 4:**
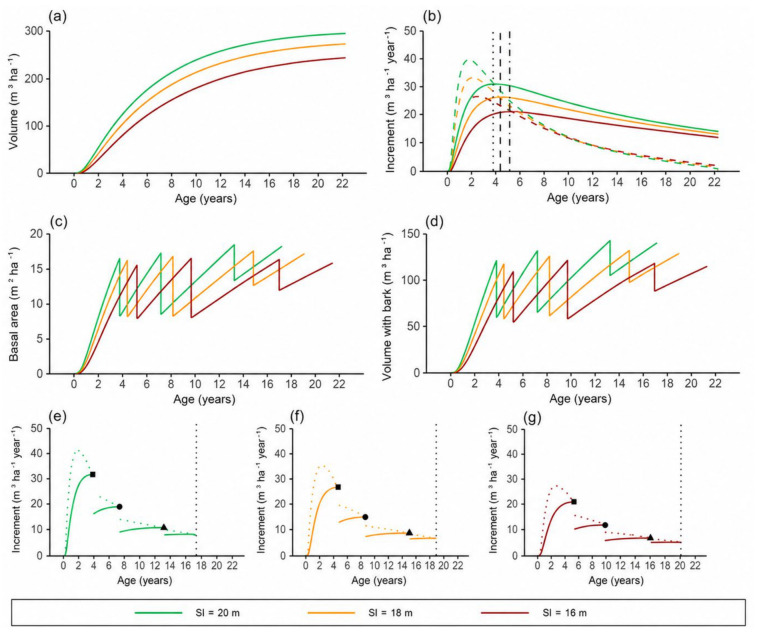
Growth and yield curves by productivity class. (**a**) represents yield and (**b**) volume increments with bark under the no-thinning scenario; (**c**) represents basal area under the thinning scenario and (**d**) volume with bark under the thinning scenario; (**e**–**g**) represent volume increments by site class under the thinning scenario in clonal teak plantations in the Eastern Brazilian Amazon. Dashed colored lines represent the current annual increment (CAI), and solid colored lines represent the mean annual increment (MAI) (graphs **b**,**e**–**g**). Black dashed vertical lines and black points indicate the moment when MAI reaches its maximum value (graphs **b**,**e**–**g**). The square, the circle, and the triangle represent the timing of the 1st, 2nd, and 3rd thinning, respectively.

The theoretical stagnation of volume growth with bark was observed at different moments throughout the rotation and varied according to site class, occurring earlier in the most productive sites, as expected (Figure 4). Based on the initial projections, technical thinning ages (TTA) of 3.8 years (≈46 months), 4.4 years (≈53 months), and 5.2 years (≈62 months) were identified for site indices of 20, 18, and 16 m, respectively (Figure 4b,e–g). When these first TTAs were reached, a 50% reduction in stand density (N) was applied, as indicated in Figure 4c. Simulations of these reductions in N resulted in the following technical thinning ages (TTA) throughout the rotation (technical rotation age—TRA):

- 1st thinning—50% reduction in N for site indices of 16, 18, and 20 m: TTA at 5.2 years (≈62 months), 4.4 years (≈53 months), and 3.8 years (≈46 months), respectively;
- 2nd thinning—50% reduction in N for site indices of 16, 18, and 20 m: TTA at 9.7 years (≈116 months), 8.2 years (≈98 months), and 7.2 years (≈86 months), respectively;
- 3rd thinning—25% reduction in N for site indices of 16, 18, and 20 m: TTA at 16.9 (≈203 months), 14.8 (≈178 months), and 13.2 years (≈158 months), respectively;
- Rotation—100% removal of the remaining N for site indices of 16, 18, and 20 m: TRA at 21.3 years (≈256 months), 19.0 years (≈228 months), and 17.1 years (≈205 months), respectively.

Table 6 presents the variables projected by the modeling system at the individual tree level for each technical thinning age (TTA) and technical rotation age (TRA), stratified by site class. The density values (N1) represent the number of remaining trees after each simulated thinning intervention. Similarly, the volumes correspond exclusively to the volumetric storage of the remaining living trees after each thinning, excluding volumes previously removed in prior interventions. Notably, the most productive site reached a projected volume at the TRA of 139.28 m^3^ ha^−1^, exceeding the averages of 128.69 m^3^ ha^−1^ and 114.32 m^3^ ha^−1^ for the medium- and low-productivity site classes, respectively. Even with the anticipation of the final harvest by 1.9 and 4.2 years in relation to the 18 m and 16 m site classes, respectively, the most productive site still prevailed in terms of volume, as well as across all TTAs. This finding highlights the strong influence of site quality on total volume production with bark, confirming that more productive sites tend to yield higher volumes.

Additionally, this pattern was also observed for diameter with bark, total height, and basal area, with the highest values recorded in the most productive site classes across all intervention ages. For the SI 16 class at the TRA, values of 37.01 cm, 19.68 m, and 15.82 m^2^ ha^−1^ were observed for diameter, total height, and basal area, respectively. In the SI 18 class, the corresponding values were 39.59 cm, 20.29 m, and 16.86 m^2^ ha^−1^, whereas the SI 20 class presented 41.11 cm, 20.73 m, and 17.52 m^2^ ha^−1^. These outcomes support the idea that more productive sites promote the development of trees with larger diameters, greater heights, and higher basal area.

**Figure 5:**
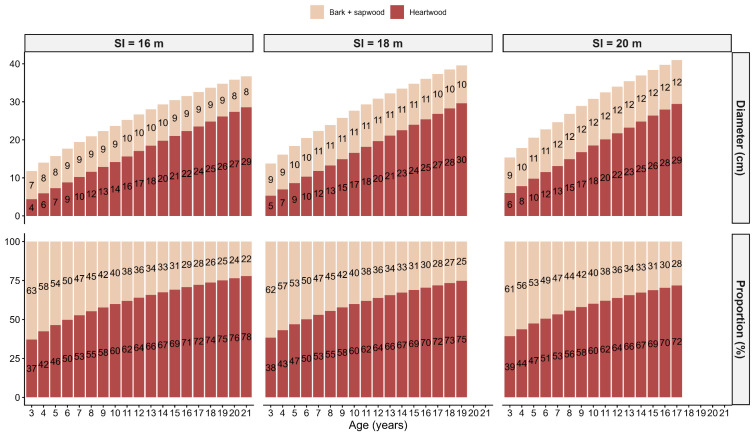
Changes in heartwood, bark, and sapwood diameter (calculated as the difference between the diameter with bark and the heartwood diameter) and the proportion between heartwood and diameter with bark, by site index (SI), in clonal teak plantations in the Eastern Amazon region of Brazil.

Figure 5 illustrates the changes in heartwood diameter and the corresponding bark + sapwood component (calculated as the difference between the bark-included diameter and the heartwood diameter) in clonal teak trees, along with the proportion of heartwood relative to the total bark-included diameter. A clear pattern of increasing heartwood proportion over time is evident. Initially, at 3 years of age, the heartwood diameter represented approximately 37% (4 cm) to 39% (6 cm) of the total bark-included diameter, depending on the respective site index (SI). This heartwood proportion increased progressively, reaching values between 72% and 78% at technical rotation ages, depending on site productivity. At these ages, the projected heartwood diameter ranged from 28.95 cm to 29.59 cm across site classes, corresponding to approximately 30 cm of heartwood diameter per tree. At technical rotation ages, the difference between the diameter with bark and the heartwood diameter ranged from approximately 8 to 12 cm across site classes, corresponding to an estimated radial thickness of bark and sapwood of approximately 4 to 6 cm.

We verified that the heartwood diameter growth rate shows an increasing trend throughout the stand’s development. In less productive sites characterized by longer rotations, the projected heartwood diameter reached approximately 29 cm and accounted for up to 78% of the total diameter with bark at 21 years of age. These results evidence the dynamics of heartwood growth in teak trees and its strong relationship with both temporal development and plantation yield characteristics.

**Figure 6:**
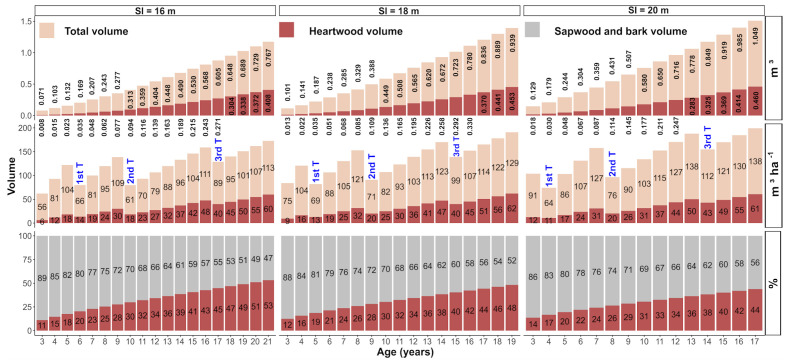
Evolution of heartwood volume at the tree level (m^3^), per unit area (m^3^ ha^−1^), and as a proportion (%) of total volume with bark, by site index (SI) in teak plantations in the Eastern Brazilian Amazon. Here, 1st T, 2nd T, and 3rd T denote the first, second, and third thinning, respectively.

In Figure 6, we assessed the amount and proportion of heartwood volume relative to total tree volume throughout the entire growth cycle of clonal teak plantations, also considering site classes. We observed that heartwood proportion was higher in the less productive site classes from 14 years of age up to the TRA. At 21 years of age, in the lowest productivity class, heartwood volume represented 53% of total volume, corresponding to 60 m^3^ ha^−1^. Comparatively, in the medium productivity class, at 19 years of age, heartwood proportion was 48% (62 m^3^ ha^−1^), whereas in the high productivity class, at 17 years of age, such proportion was 44% (61 m^3^ ha^−1^). These outcomes suggest that trees growing in less productive sites tend to develop a higher proportion of heartwood relative to their total volume when compared to trees in more productive sites.

However, when analyzing mean heartwood volume per tree, an opposite trend was observed. Better site conditions resulted in greater heartwood volume per individual tree. At 21 years of age, for instance, mean heartwood volume was 0.408 m^3^, while at 19 years it was 0.453 m^3^, and at 17 years it was 0.460 m^3^ for the 16 m, 18 m, and 20 m site classes, respectively. These results indicate that, although trees in less productive sites have a higher proportion of heartwood relative to the wood total volume, individual trees in more productive sites have a higher average heartwood volume. Therefore, higher site productivity appears to favor heartwood production at the individual-tree level, resulting in higher heartwood volumes, even with shorter rotation ages.

## 4. Discussion

### 4.1. Effect of Stand Density on Diameter and Height Projections

The number of trees proved to be a relevant and significant variable for predicting future tree diameter and height, making the modified Lundqvist–Korf-ADA base model the preferred choice for future applications and inferences related to the growth of teak trees submitted to thinning under the conditions established in the current study. The models’ suitable performance confirms that stand age and density capture the main biological processes governing teak growth, namely age-dependent growth and competition among trees. These factors are the primary determinants of diameter and height development and are directly affected by thinning operations.

This result reinforces the importance of considering silvicultural factors, such as stand density, when developing dynamic models for forest growth and yield projection [54]. In this context, to ensure efficient analysis of growth and yield in thinned teak stands, it is necessary to incorporate thinning-related information, such as covariates, in the modeling process. This allows for a better understanding of the influence of this treatment on forest variables over the rotation period. This finding is supported by the study conducted by Souza et al. [13]. These authors used the number of trees before and after thinning, as well as dominant height and thinning age as predictive covariates in basal area models for clonal teak stands, obtaining accurate results for the estimation of this variable.

Including the number of trees resulted in significant improvements in the accuracy of the projections in our study, reducing the RMSE from 0.8697 to 0.7723 cm and the RMSPE from 4.86% to 4.32% for diameter projections (Table 3). This outcome was particularly evident for diameter, since this variable is highly sensitive to stand density when compared to other dendrometric variables [55]. The most pronounced influence of thinning in teak stands is observed in diameter, and the approach adopted in the present study confirms such behavior. This improvement is particularly valuable when considering fluctuations in stand density over time, since stand density is a deterministic variable and therefore easily obtained from forest inventories [56].

The incorporation of the number of trees (N) as a covariate in dynamic projection models not only improved predictive accuracy but also contributed significantly to decision-making in the management of clonal stands of the species. Thus, the development of models including N allowed the analysis of its influence on the variation in diameter and total height of clonal teak trees. In this regard, the outcomes of including the number of trees as a covariate in dynamic models demonstrate that the reduction in tree density per hectare, caused by thinning, triggers a new growth dynamic for both variables [54]. This effect is likely due to the fact that thinning reduces intraspecific competition among trees, allowing a resumption of vigorous growth after a theoretical growth stagnation, which is essential to ensure individual timber volume increment [56,57,58]. Additionally, stand density provides a dynamic representation of competition throughout the stand’s development, reflecting the effects of both initial tree spacing and subsequent thinning operations.

### 4.2. Technical Thinning and Rotation Ages, Site Productivity and Stand Yield

The growth and productivity patterns of teak tree clones were consistent across productivity classes (Table 6 and Figure 4), with technical rotation ages ranging from 17.1 to 21.3 years and final volumes of the remaining stand ranging from 114.32 to 139.28 m^3^ ha^−1^. We particularly observed that in high-productivity sites, technical thinning and final rotation ages occurred earlier in comparison to low-productivity areas, as expected. This finding is consistent with results reported in other studies, such as those developed by Santos et al. [12] and Souza et al. [13], when assessing clonal teak plantations conducted in the Eastern Amazon, Brazil, as well as the study performed by Bermejo et al. [59], which was carried out in Costa Rica. It is worth noting that all the cited studies used whole-stand modeling (WPM).

It is important to emphasize that the technical thinning (TTA) and the rotation (TRA) ages, presented in the present study, represent theoretical values derived from continuous growth and production functions. Thus, decimal ages such as 3.8, 4.4, 5.2, 7.2, 8.2, and 9.7 years correspond to the exact mathematical points at which the optimization criteria adopted in this study were met. In operational forest management, however, thinning interventions are typically scheduled according to annual planning cycles, climatic conditions, and logistical constraints. Thus, these values should be interpreted as technical references for decision-making and may be adjusted to the nearest practical management age without substantially affecting stand productivity or heartwood production.

Our results regarding silvicultural interventions in clonal teak plantations were compared with previous studies conducted by Santos et al. [12] and Souza et al. [13], which also evaluated the very same stand from the current study. We found that the ages of the first theoretical stagnation of volume growth (3.8 to 5.2 years) are consistent with the ranges reported in the literature, particularly within the period between 3 and 8 years of age [60,61,62]. Santos et al. [11] reported a range of 3.5 to 4.2 years for the first theoretical stagnation, while Souza et al. [13] observed an interval of 4.7 to 6.3 years. Our results also showed strong consistency with the existing literature, reinforcing the conclusions of previous studies, such as those mentioned above.

Comparing our results with the study performed by Santos et al. [12], which simulated thinning practices using WPM, we observed differences of less than one year in the projected thinning intervals closer to the rotation age. While our study indicated intervals longer than 6 years between the second and third thinning, depending on site class, Santos et al. [12] reported shorter intervals of less than 5.5 years. The more widely spaced intervention intervals in our study resulted in longer rotation ages, ranging from 17.1 to 21.3 years. In contrast, Santos et al. [12] obtained shorter rotations, varying from 13.9 to 16.6 years. obtained shorter rotations, varying from 13.9 to 16.6 years. These differences suggest that factors beyond the timing of the first growth stagnation may influence the definition of subsequent thinning intervals and final rotation ages.

In addition to comparisons with modeling studies, the results obtained in this study are relevant to the silvicultural regimes typically adopted for teak in Brazil. In seed-established stands, three or four thinning operations are frequently used, with intervals ranging from four to six years and final rotations between 20 and 25 years, depending on site conditions and management objectives [12]. In this context, the thinning intervals projected in this study remain within the operationally feasible range for the species, although they indicate rotations that are comparable to or shorter than those typically adopted in seed-origin stands.

Although the ages at which growth theoretically first stagnated showed a high degree of agreement with the results of Santos et al. [12] and Souza et al. [13], the differences observed in the intervals between subsequent thinning may be related to the modeling approach adopted. While previous studies were conducted at the stand level (Whole-Stand Modeling—WPM), the present study employed an Individual-Tree Modeling (ITM) system, explicitly incorporating future stand density as a covariate in diameter and height projections. This approach allows a more detailed representation of the remaining trees’ response after each silvicultural intervention. Additionally, the current study used a longer time series, comprising ten years of monitoring, whereas Santos et al. [12] performed monitoring during seven years. These factors may have contributed to the projection of a more prolonged growth response following thinning, resulting in longer intervals between interventions and higher technical rotation ages.

Therefore, our results reinforce the consistency of these growth patterns in clonal teak plantations. The convergence between our data and previous studies strengthens confidence in the assessment and prediction of forest growth at younger ages, contributing to improved management and planning of these stands. In addition, the adoption of individual-tree modeling can provide a more detailed understanding of growth and productivity dynamics, enabling the development of more appropriate strategies for managing clonal plantations.

Based on the aforementioned, there is evidence supporting the first hypothesis, which states that production of volume without bark is enhanced by conducting three thinning operations at the times of maximum average annual growth, resulting in a final rotation cycle of less than 25 years. The clonal stands evaluated in this study reached final harvest at an age shorter than the 25-year rotation reported for seed-derived teak stands in Brazil by Bezerra et al. [63], suggesting potential for reducing the rotation age, although such differences are expected due to the particularities of the genetic material evaluated and the silvicultural practices adopted.

The differences in technical ages according to site productivity observed in our results reinforce the importance of considering site quality in forest management and in the planning of clonal teak plantations [64]. More productive sites present more favorable conditions in terms of soil fertility, structure, and drainage, as well as nutrient balance, and microclimate, providing a more suitable environment for tree growth [64]. The increase in diameter, total height, basal area, and volume in the more productive site classes indicates that these variables are interdependent and that environmental factors, such as those mentioned above, influence tree morphological development, resulting in growth characteristics that enhance productivity [65].

It is important to emphasize how relevant knowledge of the relationship between site quality and teak tree growth is for its development, as it contributes to a more rational use of natural resources, avoiding practices that may lead to soil depletion and environmental degradation [66,67]. The findings of this study highlight the importance of site conditions for volume production and tree growth in clonal teak plantations, particularly at the individual-tree level (individual tree volume). Therefore, when planning and managing teak plantations, it is essential to consider site productivity capacity to optimize production and to ensure timberwood quality through appropriate forest regulation [38].

### 4.3. Heartwood Formation in Teak and Management Implications

Heartwood formation in teak trees is influenced by several factors, such as tree age, longitudinal variation, geographic location, environmental conditions, silvicultural practices, and the thinning regime adopted [2,20,26,29,68]. The results presented in Figure 5 and Figure 6 show that heartwood formation was more strongly influenced by tree age than by site class. At the technical rotation ages, the heartwood diameter reached approximately 29–30 cm and accounted for about 72–78% of the total diameter, including bark. This behavior is expected, as heartwood gradually develops in the inner layers of the stem with increasing age [36]. This process occurs through the lignification process of older (inner) wood growth rings, turning them physiologically inactive, which represents a tree’s protective strategy against external environmental agents and pathogens [69].

Additionally, the relationship between environmental stress and heartwood formation is an important issue, although still not fully understood among researchers [70]. Under more unfavorable environmental conditions, in which plants are subjected to greater water stress and nutrient limitation, heartwood proportion may be enhanced, in some cases. This may occur because the tree faces limitations in sapwood growth, which is the living and active portion of the stem responsible for the formation of new growth layers. A particularly common stress factor in teak trees is related to dry seasons, which accelerate wood maturation and promote the heartwood formation process [71]. In addition, Kokutse et al. [72], when studying factors influencing heartwood formation in teak, found a possible relationship between dominant trees and a higher proportion of heartwood, which may also be associated with stand age.

The results obtained in the present study also show that as the intervention age increases (i.e., the more advanced the technical age at harvest), the greater the amount of heartwood found in teak trees [2]. One of the factors affecting heartwood formation is silvicultural practices, such as thinning. Moderate and heavy thinning practices tend to intensify the heartwood formation process [68]. Trees that undergo a longer growth cycle may accumulate a higher proportion of heartwood [2], especially in sites where conditions favor this process [30].

From a management perspective, these results indicate that both thinning regimes and rotation cycle duration directly influence heartwood production. Longer cycles increase the proportion of heartwood, while higher site productivity favors an increase in the absolute volume of heartwood per tree. Therefore, both factors must be considered in forestry planning aimed at maximizing the value of teak wood.

Based on this, there is evidence not to reject the second hypothesis formulated in this study, which states that the proposed individual-tree modeling system is capable of predicting heartwood formation and development in teak trees throughout the forest cycle, allowing the quantification of the stem structure that adds greater value to the species’ timber.

The ability of the modeling system to estimate diameter and heartwood volume in living trees adds substantial value to forest management practices. When combined with its capacity to simulate productivity scenarios related to thinning interventions, the individual-tree modeling system becomes a truly comprehensive and powerful tool for managing clonal teak plantations. Its application provides not only operational efficiency but also contributes to the long-term sustainability and economic success of these forest companies. This approach offers managers a more complete and accurate understanding of stand composition, enabling the identification of trees with high-quality timber potential and optimizing resource allocation during thinning and harvest operations.

## 5. Conclusions

The Lundqvist–Korf-ADA model demonstrated greater accuracy in estimating future tree diameter and height, particularly when the number of trees was included as a covariate to account for thinning. This individual-tree modeling system, specifically developed for clonal teak, proved to be a reliable tool for growth prediction. By projecting optimal technical thinning ages, the model indicated technical rotations ranging from 17.1 to 21.3 years (approximately 205 to 256 months), considering volume with bark.

A gradual increase in heartwood diameter (dh) and heartwood volume was observed based on the model outputs, reaching up to 72–78% (29–30 cm) and 44–53% (0.408–0.460 m^3^ tree^−1^) at rotation ages, respectively, highlighting the potential for high-quality timber production at more advanced ages. By generating more precise and detailed predictions, this approach strengthens strategic decision-making in the management of clonal teak plantations, allowing for the optimization of thinning and harvest cycles to maximize both wood production and quality.

## Figures and Tables

**Table 1:** Growth models for diameter with bark and total height using ADA and GADA, selected for fitting individual-tree data in clonal teak plantations in the Eastern Brazilian Amazon.

Model	Base Model	Parameters Related to Target Variable X	Initial Solution for X with Y_0_ and t_0_	Dynamic Equation
M1	Lundqvist–Korf ${Y=\;A\;exp}^{\left(-B\;t^{\;-\Gamma }\right)}$	B=X	$X_{0}=-Ln\;\left(\frac{Y_{0}}{b_{1}}\right){t_{0}}^{b_{3}}$	${Y=b}_{1}\;{exp}^{\left[Ln\;\left(\frac{Y_{0}}{b_{1}}\right)\;{\left(\frac{t_{0}}{t_{1}}\right)}^{b_{3}}\right]}$
M2	Hossfeld $Y\;=\;\frac{A}{1\;+\;B\;t^{-\Gamma }}$	B=X	$X_{0}={{\;t}_{0}}^{-b_{3}}\;\left(\frac{b_{1}}{Y_{0}}-1\right)$	${Y=b}_{1}/\left[1-\left(1-\frac{b_{1}}{Y_{0}}\right){\left(\frac{t_{0}}{t_{1}}\right)}^{b_{3}}\right]$
M3	Cieszewski $Y\;=\;\frac{B\;t^{\;\Gamma }}{t^{\;\Gamma \;}+\;A}$	A=B+X $B=\frac{A}{X}$	$X_{0}=Y_{0\;}-{\;b}_{1}+\sqrt{{\left(Y_{0}-b_{1}\right)}^{2}+2{\;Y}_{0}{\;exp}^{\left(\frac{b_{1}}{t_{0}^{\;b_{3}}}\right)}}$	${Y=Y}_{0}\left[\frac{{t_{1}}^{b_{3}}({t_{0}}^{b_{3}}X_{0})\;}{{t_{0}}^{b_{3}}({t_{1}}^{b_{3}}X_{0})\;}\right]$
M4	Lundqvist–Korf ${Y\;=\;A\;exp}^{\left(-B\;t^{-\Gamma }\right)}$	$A={exp}^{\left(X\right)}$ $B={(b}_{1}+b_{2})/X$	$X_{0}\;=\;\frac{1}{2}\;{t_{0}}^{-b_{3}}\left[b_{1}+\;{t_{0}}^{b_{3}}\;Ln{(Y}_{0})\;\pm \;\sqrt{{{4\;b_{2}t}_{0}}^{b_{3}}+\;L_{0}}\;\right]$ where $L_{0}\;=\;{\left(-b_{1}\;{-t_{0}}^{b_{3}}Ln(Y_{0})\right)}^{2}$	${Y=exp}^{{(X}_{0})}{\;exp}^{\left[-\left(\frac{b_{1+}b_{2}}{X_{0}}\right)\;{t_{1}}^{{-b}_{3}}\right]\;\;}$

Y and Y_0_: variables of interest at ages t_0_ and t_1_, respectively; t, t_0_, and t_1_: tree ages (years); X: theoretical, unobservable and non-quantifiable variable; X_0_: solution of X for diameter, height, and initial age; A, B, and Γ: base model parameters; b_1_, b_2_, and b_3_: global parameters of the dynamic equations. Source: [36,37,38,40].

**Table 2:** Descriptive statistics of tree-level variables in clonal teak plantations in the Eastern Brazilian Amazon.

Variables	Fitting Dataset				Validation Dataset			
	Min.	Max.	Mean	SD	Min.	Max.	Mean	SD
t	2	12	5.04	2.06	2	10	5.02	1.95
dbh_wb_	3.80	38.64	17.90	5.16	4.30	34.54	18.09	4.42
dbh_h_	0.77	24.68	8.55	3.74	1.33	20.26	8.60	3.29
ht	3.30	21.50	13.19	2.68	5.10	19.70	13.41	2.30
N	120	960	571.5	218.46	100	860	582.8	213.56

t: age (years); dbh_wb_: diameter at breast height with bark (cm); dbh_h_: diameter at breast height of heartwood (cm); ht: total height (m); N: number of trees (trees ha^−1^); SD: standard deviation.

**Table 3:** Estimators and goodness-of-fit statistics of ADA and GADA models for estimating future diameter with bark in clonal teak plantations in the Eastern Brazilian Amazon.

Model	Parameter	SD	$r_{\hat{y}y}$	RMSE	RMSPE	AIC
M1	b_1_ = 97.388 **	4.179	0.986	0.870	4.859	17,054.08
	b_3_ = 0.330 **	0.007				
M2	b_1_ = 49.769 **	1.027	0.984	0.924	5.164	17,865.77
	b_2_ = 0.909 **	0.009				
M3	b_1_ = 90.737 **	19.024	0.983	0.960	5.366	18,378.37
	b_2_ = 1.279 **	0.319				
	b_3_ = 0.779 **	0.013				
M4	b_1_ = −27,504.895 **	138.904	0.987	0.901	5.037	17,533.89
	b_2_ = 108,345.210 **	542.425				
	b_3_ = 0.49702 **	0.003				
M5 ^#^	b_1_ = 1456.516 **	90.988	0.989	0.772	4.315	15,473.19
	b_11_ = −0.557 **	0.009				
	b_3_ = 0.595 **	0.009				

b_i_: model parameters; SD = standard deviation of the parameters; RMSE: root mean square error (cm); RMSPE: root mean square percentage error (%); $r_{\hat{y}y}$: correlation coefficient between observed and predicted values; AIC: Akaike Information Criterion; ^#^: model modified with the covariate N_1_. **: Regression parameters were significant at the 1% level (*p* < 0.01) according to Student’s *t*-test.

**Table 4:** Estimators and goodness-of-fit statistics of ADA and GADA models for estimating future total height in clonal teak plantations in the Eastern Brazilian Amazon.

Model	Parameters	SD	$r_{\hat{y}y}$	RMSE	RMSPE	AIC
M1	b_1_ = 24.624 **	0.686	0.949	0.831	5.755	5392.55
	b_3_ = 0.621 **	0.023				
M2	b_1_ = 20.553 **	0.313	0.949	0.832	5.764	5396.57
	b_2_ = 1.130 **	0.025				
M3	b_1_ = 22.781 **	2.168	0.946	0.865	5.989	5568.54
	b_2_ = 1.730 **	0.236				
	b_3_ = 0.922 **	0.025				
M4	b_1_ = −29,332.246 **	396.764	0.949	0.831	5.755	5394.64
	b_2_ = 94,040.480 **	1263.740				
	b_3_ = 0.618 **	0.008				
M5 ^#^	b_1_ = 66.390 **	7.569	0.951	0.820	5.678	5335.73
	b_11_ = −0.183 **	0.019				
	b_3_ = 0.790 **	0.033				

b_i_: model parameters; SD = standard deviation of the parameters; RMSE: root mean square error (m); RMSPE: root mean square percentage error (%); $r_{\hat{y}y}$: correlation coefficient between observed and predicted values; AIC: Akaike Information Criterion; ^#^: model modified with the covariate N_1_. **: Regression parameters were significant at the 1% level (*p* < 0.01) according to Student’s *t*-test.

**Table 5:** Results of the TOST equivalence test used for validation of the selected models for estimating diameter at breast height with bark (dbh) and total height (ht) in clonal teak plantations in the Eastern Brazilian Amazon.

Model	Parameters	Similarity Region	Confidence Interval	TOST Result
M5—dbh	Intercept	(17.87; 18.37)	(18.04; 18.12)	Reject
	Slope	(0.75; 1.25)	(0.98; 1.00)	Reject
M5—ht	Intercept	(14.22; 14.72)	(14.35; 14.48)	Reject
	Slope	(0.75; 1.25)	(0.91; 0.98)	Reject

The values are presented in the format (Lower; Upper). “Reject” indicates rejection of the null hypothesis of non-equivalence in the TOST procedure, confirming statistical equivalence between the observed and estimated values.

**Table 6:** Projected variables for the remaining stand after each simulated thinning intervention and at the technical rotation age, by site class, generated by the individual-tree modeling system in clonal teak stands in the Eastern Brazilian Amazon.

Variable	TTA			TRA	TTA			TRA	TTA			TRA
	1st	2nd	3rd		1st	2nd	3rd		1st	2nd	3rd	
	SI = 16 m				SI = 18 m				SI = 20 m			
Age	5.2	9.7	16.9	21.3	4.4	8.2	14.8	19	3.8	7.2	13.2	17.1
N_b_	784	392	196	147	739	369	183	137	709	354	177	132
N_1_	392	196	147	147	369	183	137	137	354	177	132	132
ht_1_	12.29	15.46	18.47	19.68	12.69	15.82	19.00	20.29	13.09	16.24	19.43	20.73
dbh_1wb_	16.04	23.14	32.45	37.01	16.86	24.18	34.47	39.59	17.26	24.95	35.70	41.11
dbh_1h_	7.55	13.71	23.39	28.95	7.53	13.53	23.63	29.59	7.40	13.40	23.53	29.57
G_1wb_	8.14	8.36	12.24	15.82	8.49	8.55	12.89	16.86	8.58	8.82	13.33	17.52
V_1wb_	55.55	59.56	88.97	114.32	59.35	62.91	98.21	128.69	61.19	66.69	105.32	139.28
V_1h_	10.24	17.55	39.87	61.54	10.22	16.74	39.47	62.07	10.05	16.61	38.89	61.35

Age = technical intervention age expressed in decimal years. For operational interpretation, the values of 3.8, 4.4, 5.2, 7.2, 8.2, and 9.7 years correspond approximately to 46, 53, 62, 86, 98, and 116 months, respectively; TTA = technical thinning age (years); TRA = technical rotation age (years); SI = site index (m); N_b_ = number of trees before thinning (trees ha^−1^); N_1_ = number of trees after thinning (trees ha^−1^); ht_1_ = total height after thinning (m); dbh_1wb_ = diameter at breast height with bark (cm) after thinning; dbh_1h_ = heartwood diameter at breast height (cm) after thinning; G_1wb_ = basal area (m^2^ ha^−1^) after thinning; V_1wb_ = total volume with bark (m^3^ ha^−1^) after thinning; V_1h_ = heartwood volume (m^3^ ha^−1^) after thinning.

## Data Availability

The data are not publicly available due to the policy of the company (Tietê Agrícola Ltda.) that owns the teak plantations.

## Abbreviations

The following abbreviations are used in this manuscript:

ITM	Individual-tree modeling
TTA	Technical thinning age
TRA	Technical rotation age
MAI	Mean annual increment
CAI	Current annual increment
dbh_wb_	Diameter at breast height with bark
dbh_h_	Diameter at breast height of heartwood
ht	Total height
dh	Dominant height

## References

[1] Pachas A.N.A., Sakanphet S., Midgley S., Dieters M. (2019). Teak (*Tectona grandis*) silviculture and research: Applications for smallholders in Lao PDR. Aust. For..

[2] Berrocal A., Gaitan-Alvarez J., Moya R., Fernández-Sólis D., Ortiz-Malavassi E. (2020). Development of heartwood, sapwood, bark, pith and specific gravity of teak (*Tectona grandis*) in fast-growing plantations in Costa Rica. J. For. Res..

[3] Kusbach A., Šebesta J., Meason D.F., Mikita T., Meyrat A.M.C., Janata P., Maděra P., Hybler V., Smola M. (2021). Site-specific approach to growth assessment and cultivation of teak (*Tectona grandis*) in Nicaraguan dry tropics. For. Ecol. Manag..

[4] Cao Q.V. (2014). Linking individual-tree and whole-stand models for forest growth and yield prediction. For. Ecosyst..

[5] Ferraz Filho A.C., Scolforo J.R.S., Oliveira A.D., Mello J.M. (2015). Modeling growth and yield of loblolly pinestands under intensive management. Pesqui. Agropecu. Bras..

[6] Davis L., Johnson K., Bettinger P., Howard T. (2005). Forest Management: To Sustain Ecological, Economic, and Social Values.

[7] Carrijo J.V.N., Ferreira A.B.F., Ferreira M.C., Aguiar M.C., Miguel E.P., Matricardi E.A.T., Rezende A.V. (2020). The growth and production modeling of individual trees of *Eucalyptus urophylla* plantations. J. For. Res..

[8] Miguel E.P., Machado S.A., Figueiredo Filho A., Arce J.E. (2010). Using the Wweibull function for prognosis of yield by diameter class in *Eucalyptus urophylla* stands. Cerne.

[9] Weiskittel A.R., Hann D.W., Kershaw J.A., Vanclay J.K. (2011). Forest Growth and Yield Modeling.

[10] Castro R.V.O., Soares C.P.B., Martins F.B., Leite H.G. (2013). Crescimento e produção de plantios comerciais de eucalipto estimados por duas categorias de modelos. Pesqui. Agropecu. Bras..

[11] Tondjo K., Brancheriau L., Sabatier S., Kokutse A.D., Kokou K., Jaeger M., de Reffye P., Fourcaud T. (2018). Stochastic modelling of tree architecture and biomass allocation: Application to teak (*Tectona grandis* L. f.), a tree species with polycyclic growth and leaf neoformation. Ann. Bot..

[12] Santos M.L., Miguel E.P., Santos C.R.C., Souza H.J., Martins W.B.R., Lima M.D.R., Arce J.E., Silva J.N.M. (2022). Forecasting production in thinned clonal stands of *Tectona grandis* in Eastern Amazonia. For. Syst..

[13] Souza H.J., Miguel E.P., Nascimento R.G.M., Cabacinha C.D., Rezende A.V., Santos M.L. (2022). Thinning-response modifier term in growth models: An application on clonal *Tectona grandis* Linn F. stands at the amazonian region. For. Ecol. Manag..

[14] Nogueira G.S., Leite H.G., Campos J.C.C., Carvalho A.F., Souza A.L. (2005). delo de distribuição diamétrica para povoamentos de *Eucalyptus* sp. submetidos a desbaste. Rev. Árvore.

[15] Leite H.G., Nogueira G.S., Campos J.C.C., Takizawa F.H., Rodrigues F.L. (2006). Um modelo de distribuição diamétrica para povoamentos de *Tectona grandis* submetidos a desbaste. Rev. Árvore.

[16] Madi J.P.S., Vendruscolo D.G.S., Silva C.A., Carvalho M.P.L.C., Carvalho S.P.C. (2017). Univariate models to represent the diametric distribution of thinned stand of *Tectona grandis* Linn.F. Adv. For. Sci..

[17] Medeiros R.A., de Paiva H.N., Leite H.G., Salles T.T., Araújo Júnior C.A., Dávila F.S. (2017). Technical age for the first thinning of teak stands in different spacings. Sci. For..

[18] Leite H.G., Oliveira-Neto R.R.D., Monte M.A., Fardin L., Alcântara A.M.D., Binoti M.L.M.D.S., Castro R.V.O. (2011). Modelo de afilamento de cerne de *Tectona grandis* L.f. Sci. For..

[19] Leite H.G., Marques da Silva M.L., Binoti D.H.B., Fardin L., Takizawa F.H. (2011). Estimation of inside-bark diameter and heartwood diameter for *Tectona grandis* Linn. trees using artificial neural networks. Eur. J. For. Res..

[20] Fernández-Sólis D., Berrocal A., Moya R. (2018). Heartwood formation and prediction of heartwood parameters in Tectona grandis L.f. trees growing in forest plantations in Costa Rica. Bois Forêts Trop..

[21] Moya R., Gaitán-Álvarez J., Ortiz-Malavassi E., Berrocal A., Fernández-Sólis D. (2020). Equations for predicting heartwood merchantable volume and tradable sawlog in *Tectona grandis*. J. Trop. For. Sci..

[22] Porté A., Bartelink H.H. (2002). Modelling mixed forest growth: A review of models for forest management. Ecol. Model..

[23] Pretzsch H., Grote R., Reineking B., Rötzer T., Seifert S. (2008). Models for Forest Ecosystem Management: A European Perspective. Ann. Bot..

[24] Twery M.J., Weiskittel A.R. (2013). Forest-management modelling. Environmental Modelling: Finding Simplicity in Complexity.

[25] Seppänen P., Mäkinen A. (2020). Comprehensive yield model for plantation teak in Panama. Silva Fenn..

[26] Moya R., Bond B., Quesada H. (2014). A review of heartwood properties of *Tectona grandis* trees from fast-growth plantations. Wood Sci. Technol..

[27] Yang B., Jia H., Zhao Z., Pang S., Cai D. (2020). Horizontal and Vertical Distributions of Heartwood for Teak Plantation. Forests.

[28] Wang X., Wang C., Zhang Q., Quan X. (2010). Heartwood and sapwood allometry of seven Chinese temperate tree species. Ann. For. Sci..

[29] Kokutse A.D., Baillères H., Stokes A., Kokou K. (2004). Proportion and quality of heartwood in Togolese teak (*Tectona grandis* L.f.). For. Ecol. Manag..

[30] Tewari V.P., Mariswamy K.M. (2013). Heartwood, sapwood and bark content of teak trees grown in Karnataka, India. J. For. Res..

[31] IBGE (2012). Manual Técnico da Vegetação Brasileira.

[32] Santos H.G., Jacomine P.K.T., Anjos L.H.C., Oliveira V.A., Lumbreras J.F., Coelho M.R., Almeida J.A., Araújo Filho J.C., Lima H.N. (2025). Sistema Brasileiro de Classificação de Solos.

[33] Alvares C.A., Stape J.L., Sentelhas P.C., Gonçalves J.L.M., Sparovek G. (2013). Köppen’s climate classification map for Brazil. Meteorol. Z..

[34] INMET (2023). Normais Climatológicas do Brasil: 1991–2020.

[35] Haglöf (2007). Vertex IV and Transponder T3 Manual.

[36] Tewari V.P., Álvarez-González J.G., García O. (2014). Developing a dynamic growth model for teak plantations in India. For. Ecosyst..

[37] Cieszewski C.J., Strub M. (2008). Generalized Algebraic Difference Approach Derivation of Dynamic Site Equations with Polymorphism and Variable Asymptotes from Exponential and Logarithmic Functions. For. Sci..

[38] Cieszewski C.J., Bailey R.L. (2000). Generalized Algebraic Difference Approach: Theory based derivation of dynamic site equations with polymorphism and variable asymptotes. For. Sci..

[39] Kiviste A., Kiviste K. (2009). Algebraic Difference Equations of stand height, diameter, and volume depending on stand age and site factors for Estonian State Forests. Math. Comput. For. Nat.-Resour. Sci..

[40] Burkhard H.E., Tomé M. (2012). Modeling Forest Trees and Stands.

[41] Palahí M., Tomé M., Pukkala T., Trasobares A., Montero G. (2004). Site index model for *Pinus sylvestris* in north-east Spain. For. Ecol. Manag..

[42] Richards F.J. (1959). A flexible growth function for empirical use. J. Exp. Bot..

[43] Santos M.L., Miguel E.P., Nappo M.E., Souza H.J., Santos C.R.C., Silva J.N.M., Matricardi E.A.T. (2023). Approaches to forest site classification as an indicator of teak volume production. Forests.

[44] Santos M.L.d., Miguel E.P., Biali L.J., Souza H.J.d., Santos C.R.C.d., Matricardi E.A.T. (2023). The Effect of Age on the Evolution of the Stem Profile and Heartwood Proportion of Teak Clonal Trees in the Brazilian Amazon. Forests.

[45] Parresol B.R. (1999). Assessing tree and stand biomass: A review with examples and critical comparisons. For. Sci..

[46] Akaike H. (1978). On the likelihood of a time series model. Statistician.

[47] Shapiro S.S., Wilk M.B. (1965). An analysis of variance test for normality. Biometrika.

[48] R Core Team (2023). R: A Language and Environment for Statistical Computing.

[49] Gregoire T.G., Schabenberger O., Barrett J.P. (1995). Linear modelling of irregularly spaced, unbalanced longitudinal data from permanent-plot measurements. Can. J. For. Res..

[50] García O., Burkhart H.E., Amateis R.L. (2011). A biologically-consistent stand growth model for loblolly pine in the Piedmont physiographic region, USA. For. Ecol. Manag..

[51] Robinson A.P., Duursma R.A., Marshall J.D. (2005). A regression-based equivalence test for model validation: Shifting the burden of proof. Tree Physiol..

[52] Leuschner W.A. (1990). Forest Regulation, Harvest Scheduling, and Planning Techniques.

[53] Campos J.C.C., Leite H.G. (2017). Mensuração Florestal: Perguntas e Respostas.

[54] Wirabuana P.Y.A.P., Hendrati R.L., Baskorowati L., Susanto M., Mashudi, Sulistiadi H.B.S., Setiadi D., Sumardi, Alam S. (2022). Growth performance, biomass accumulation, and energy production in age series of clonal teak plantation. For. Sci. Technol..

[55] Seta G.W., Widiyatno, Hidayati F., Na’iem M. (2021). Impact of thinning and pruning on tree growth, stress wave velocity, and pilodyn penetration response of clonal teak (*Tectona grandis*) plantation. For. Sci. Technol..

[56] Ferraz Filho A.C., Mola-Yudego B., González-Olabarria J.R., Scolforo J.R.S. (2018). Thinning regimes and initial spacing for Eucalyptus plantations in Brazil. An. Acad. Bras. Ciências.

[57] Nogueira G.S., Marshall P.L., Leite H.G., Campos J.C.C. (2015). Thinning intensity and pruning impacts on *Eucalyptus* plantations in Brazil. Int. J. For. Res..

[58] Sadono R. (2017). Temporary site index for two-invented teak clones with generative regeneration in the State Forestland in East Java, Indonesia. Adv. Environ. Biol..

[59] Bermejo I., Cañellas I., San Miguel A. (2004). Growth and yield models for teak plantations in Costa Rica. For. Ecol. Manag..

[60] Cañadas-L Á., Andrade-Candell J., Domínguez-A J.M., Molina-H C., Schnabel-D O., Vargas-Hernández J.J., Wehenkel C. (2018). Growth and yield models for teak planted as living fences in coastal Ecuador. Forests.

[61] Kollert W., Kleine M. (2017). The Global Teak Study: Analysis, Evaluation and Future Potential of Teak Resources.

[62] Vendruscolo D.G.S., Chaves A.G.S., Medeiros R.A., Silva R.S., Souza H.S., Drescher R., Leite H.G. (2017). Estimativa da altura de árvores de *Tectona grandis* L.f. utilizando regressão e redes neurais artificiais. Nativa.

[63] Bezerra A.F., Milagres F.R., Silva M.L., Leite H.G. (2011). Análise da viabilidade econômica de povoamentos de *Tectona grandis* submetidos a desbastes no Mato Grosso. Cerne.

[64] Santos M.L., Miguel E.P., Silva J.N.M., Santos C.R.C., Lima M.D.R., Costa B.C., Costa L.R.R., Martins W.B.R., Raddatz D.D., Rosa R.C. (2022). Spatial variability of the productive capacity of teak (*Tectona grandis* Linn F.) plantations in the eastern Amazonia. Aust. J. Crop Sci..

[65] Hlaing Z.C., Teplyakov V.K., Thant N.M.L. (2014). CrossRef citations to date 3 Altmetric Listen Original Articles Influence of climate factors on tree-ring growth in teak (*Tectona grandis* L. f.) plantations in the Bago Yoma Range, Myanmar. For. Sci. Technol..

[66] Watanabe Y., Owusu-Sekyere E., Masunaga T., Buri M.M., Oladele O.I., Wakatsuki T. (2010). Teak (*Tectona grandis*) growth as influenced by soil physicochemical properties and other site conditions in Ashanti region, Ghana. J. Food Agric. Environ..

[67] Preechamart S., Pumijumnong N., Payomrat P., Buajan S. (2018). Variation in climate signals in teak tree-ring chronologies in two different growth areas. Forests.

[68] Pérez D., Kanninen M. (2005). Effect of thinning on stem form and wood characteristics of teak (*Tectona grandis*) in a humid tropical site in Costa Rica. Silva Fenn..

[69] Bamber R.K. (1976). Heartwood, its function and formation. Wood Sci. Technol..

[70] Luo B., He R., Yang Y. (2018). A review of physiological function of sapwood and formation mechanism of heartwood. J. Beijing For. Univ..

[71] Pérez Cordero L.D., Kanninen M. (2003). Heartwood, sapwood and bark content, and wood dry density of young and mature teak (*Tectona grandis*) trees grown in Costa Rica. Silva Fenn..

[72] Kokutse A.D., Stokes A., Kokutse N.K., Kokou K. (2010). Which factors most influence heartwood distribution and radial growth in plantation teak?. Ann. For. Sci..

